# Cultural communication in museums: A perspective of the visitors experience

**DOI:** 10.1371/journal.pone.0303026

**Published:** 2024-05-09

**Authors:** Pengpeng Li

**Affiliations:** Department of Shiliangcai Journalism and Communication School, Zhejiang Sci-Tech University, Hangzhou, Zhejiang, China; PLoS ONE, UNITED STATES

## Abstract

As museums shift their responsibilities and functions towards audience-centered approaches, research on exploring museum cultural communication strategies through visitor experiences has gained increasing attention from both academia and industry. This study focuses on the newly opened Nan Song Deshou Palace Relics Site Museum in Hangzhou, China, completed at the end of 2022, and its visitors. Data were collected through on-site surveys and in-depth interviews. The research findings indicate that the current motivations of museum visitors manifest primarily in three forms: knowledge exploration, social interaction, and psychological restoration. After evaluating the existing museum service quality based on the field of experiential value in marketing management, two main issues and features were identified. The issues include sub-optimal visitor pathways and layout, dissatisfaction with staff services, and shortcomings in promotion and communication. The overall cultural learning and interactive experience for the entire visitor base also require improvement. The features are characterized by differentiated cultural and creative consumption in the museum and the emergence of interrelated consumer demands. Based on these findings, the study provides targeted recommendations for future museum construction and communication strategies.

## Introduction

Museums initially took the form of libraries, primarily serving the functions of collecting, researching, and preserving knowledge. In the mid-nineteenth century, the International Council of Museums was established in Paris, defining museums as "non-profit permanent institutions serving society and its development, open to the public, and dedicated to the collection, preservation, research, communication, and display of tangible and intangible assets related to humanity and its environment." [1] Following the impact of social movements such as the civil rights movement, women’s movement, and counterculture movement on democratic and multicultural values in the 1970s, the United States began considering audience diversity and the social and multicultural landscape in museums, actively enhancing museums’ accessibility to visitors [2]. Since then, museums have shifted from being perceived as "temples" inaccessible to the common people to becoming "public forums" [3]. Their management philosophy has gradually transitioned from focusing primarily on "things" (collection, exhibition, research, etc.) to centering on "people" (experience, learning, leisure, entertainment, etc.) [4]. In 2022, the International Council of Museums updated its definition of museums, placing significant emphasis on museums’ crucial responsibilities and missions in satisfying diverse public experiences and promoting cultural exchange [5]. Against this background and development trend, the focus of museum research has shifted from the attention on collections, environmental arrangements, and the intrinsic value of objects to how museum facilities and setups can meet the diverse needs of visitors and enhance their service experiences.

This study posits that the shifts in the concept, functions, and research focus of museums indicate an increasing awareness that visitors’ motivations, experiential encounters, autonomous dissemination intentions, and behavioral practices can influence the operational management of museums and the effectiveness of cultural dissemination at the regional level to varying degrees. Therefore, a more detailed exploration of museum functions, service development, and the external communication pathways of culture from the perspective of audience experience is crucial for enhancing museum management efficiency and fostering broader cultural communication and understanding among the general public. In contemporary society, where the contest for international discourse increasingly relies on each country’s cultural soft power, the preservation and exhibition of cultural heritage hold significant value for socio-economic, political, and international communication [6,7]. Museums, as domains of cultural heritage preservation, have garnered widespread attention for their role in cultural construction and communicative value. They are considered influential in shaping public will, cultural identity formation, fostering national cultural confidence, and serving as important mediums for intercultural communication [8–10]. In the era of globalization, modern museums simultaneously bear multiple social functions, including the inheritance of national and urban culture, cultural brand construction, image presentation, improvement of people’s livelihood, and international exchange [11,12]. They play a crucial role in educating people to shape national identity, showcasing and protecting cultural heritage, promoting cultural research, and facilitating international exchange and cooperation [13]. With the support of government policies and funding in China, the number of museums has seen significant growth since 2007, reflecting the recognition by national and municipal administrators of the vital value of local cultural heritage preservation, inheritance, dissemination, and the role of museums in national cultural soft power construction. Goode (1891) emphasized that museums should serve as sources for public cultural exchange, intellectual stimulation, and the genesis of new social ideas, thereby achieving their cultural exchange and dissemination objectives [14]. However, discussions regarding Chinese museums, both domestically and internationally, are still in their infancy [13]. Moreover, in the limited research available, there is a notable lack of discussion on aspects such as cultural perceptions, acceptance, service experiences, and dissemination behaviors of museum visitors [15].

Scholars have provided comprehensive discussions on various aspects of museums, including their public communication functions [16], educational functions [17], the maintenance and dissemination of national memory [18], technological usage [19], media image presentation [20,21], and cultural narrative forms [15,22]. These discussions primarily rely on qualitative discourse analysis as the main research method. In contrast, this study differs in its approach and methodology from discussions that primarily focus on the cultural functionalities of museums. Instead, it explores how museum construction, functional zoning, service support, and cultural narratives create, satisfy, and present spaces and needs within the context of the audience experience. The research focuses on investigating the motivations and types of museum visitors. It aims to explore the ways in which museum construction, in actuality, creates space and conditions for public communication, cultural inheritance or dissemination, and economic development. Additionally, the study delves into how visitors perceive and accept the cultural information conveyed by museums and examines the factors influencing their autonomous dissemination intentions regarding the museum and the culture displayed within it.

To better address the aforementioned questions, this study has chosen a local museum that prominently showcases specific historical and urban cultural aspects—the Nan Song Deshou Palace Relics Site Museum in Hangzhou, China. The primary rationale for this selection lies in the recent strong advocacy by the Chinese government to promote traditional culture, establish distinctive cultural brands for cities, and narrate Chinese stories from a Chinese perspective and voice. Under such policy directives, cities are committed to exploring and developing local cultures, actively creating innovative forms of communication to enhance urban cultural visibility and dissemination effectiveness. Museums, as crucial mediums for concentrating the exhibition and preservation of culture and promoting cultural dissemination [23], have garnered attention from urban administrators and relevant cultural and tourism authorities. Over the past twenty years, China has constructed thousands of museums [24]. Zhang & Courty (2022) posit that museums are typically invested in and constructed as part of significant urban or regional infrastructure. These infrastructures often include other cultural facilities such as libraries, theaters, cultural centers, and tourism-related infrastructure, and may belong to more extensive urban revitalization and historical archaeological site restoration projects [25]. Hangzhou, being a traditional cultural tourism city, has been actively seeking to stimulate local economic development by expanding the tourism industry, promoting museums, and their cultural heritage as crucial tourist attractions [26].

Among the rapidly growing various types of local museums, a notable cultural project, the Nan Song Deshou Palace Relics Site Museum, has garnered widespread attention from media and the public in Hangzhou over the past two years. Officially opened to the public at the end of 2022, the museum serves as a representative of local culture, focusing on showcasing the architecture and aesthetic lifestyle of the Nan Song period in China. It has not only gained attention from local residents and sparked lively discussions within social groups in Hangzhou, but has also been recognized by the Chinese government and media as a significant project showcasing China’s "harmony" culture, the cultural temperament of Hangzhou, and its cultural traditions. However, since the museum is still in the early stages of operation, there is a lack of research on its operational and cultural dissemination effectiveness. This study contends that, as the first concentrated display of Nan Song culture in China, discussions on its operational philosophy, cultural communication strategies, and effects can provide immediate practical insights for the museum’s sustained development. Additionally, it can extend discussions on the operational management and cultural dissemination pathways of other local museums. Simultaneously, the study aims to provide insights for cross-cultural exchange and cooperation.

Subsequently, this study conducted a detailed literature review on museum visitor research and museum experiences. The aim was to explore and establish the theoretical foundation and analytical framework for this research. Following that, a more in-depth analysis of museum visitors and their experiences was undertaken. Firstly, the study examined the types of visitors currently attracted to the museum, their primary motivations for visiting, and whether there are differences compared to previous literature or other types of museum visitors. This is crucial for a comprehensive understanding of the diverse profiles of museum visitors. Secondly, the study evaluated whether the current construction and services of the museum meet the expectations and needs of its visitors. It delved into how the museum achieves or encounters obstacles in its roles of public communication, cultural inheritance or dissemination, and economic promotion. This analysis is of significant importance for gaining insights into the current operational management status of the museum, making targeted improvements to museum operations and communication strategies, and enhancing its effectiveness in cultural dissemination.

## Literature review

### Visitors’ studies

Museum visitor studies can be traced back to as early as 1884 when the Liverpool Museum in the United Kingdom first observed the exhibition behavior of museum visitors. In the early 20th century, museum visitor research took a significant turn with Benjamin Gilman’s exploration of the visitor experience at the Boston Museum of Fine Arts in 1916 [27]. Following this, Melton’s research on museum visitors in the United States brought attention to the phenomenon and issues of "museum fatigue" among visitors [28]. Since then, the psychological and behavioral study of museum visitors has gained widespread attention from scholars across various fields.

As the study of museum visitors gradually gained attention in academia, its definition has been continuously updated and refined. In 1974, the American Association of Museums (AAM) formally established the "Committee of Audience Research and Evaluation" (CARE), interpreting "visitor studies" as the "systematic collection of information from actual and potential visitors to museums, and the use of this information in the planning and execution of activities related to the public to enhance the public’s experiences [29]."

In 2006, Ellen Giusti, an expert in museum evaluation, emphasized at the annual meeting of the American Association of Museums that museum visitor studies had not received the attention it deserved from academia and museum management. She called for placing visitor studies at the core of exhibition and activity planning, stating that such studies could directly impact the museum’s operations, services, education, and exhibitions. Giusti urged a more focused and systematic analysis of visitor descriptive, contextual, and psychological characteristics, aiming to facilitate effective management and external promotion [30]. In terms of the importance of visitor studies for museum development and marketing, it is considered a crucial type of social research. It can assist museum decision-makers, public service providers, exhibition planners, and event organizers in breaking away from the constraints of tradition, common knowledge, media opinion, and personal experience, thereby enabling a more logical and systematic approach to comprehensively understanding visitor psychological and behavioral characteristics, leading to precise positioning, planning, and communication. Looking into the future of museum visitor studies, considerations should include the uniqueness of each museum, exploring the diversity of museum learning paths, investigating the similarities and differences in short-term and long-term learning effects of museum visits, analyzing the role of museum learning in social life, discerning the differences in the learning processes and effects among different groups (such as students, families, and seniors), examining the variations in research methods and tools at different levels of museum execution, and enhancing interdisciplinary dialogue and research exchange [31].

In recent years, as management and researchers have increasingly focused on museum visitors, museums have shifted their operational strategies from being primarily focused on "objects" (collection, exhibition, research, etc.) to emphasizing strategies centered around "people" (experience, learning, leisure, and entertainment) and their effects [4]. Annis proposed that visitors in the museum environment typically exhibit three levels of engagement: the "dream space" of communication at the sensory and semi-rational level, the "reality space" of physical activities, and the "cognitive space" where they respond to their own rational thinking within the museum’s established programs [32]. Building on this, Graburn suggested that visitors in the museum environment can experience reverence, association, and educational experiences [33]. Kaplan and other scholars emphasized visitors’ needs for "relieving psychological stress" and "gaining energy" during their visits [34]. Falk & Dierking introduced a multidisciplinary approach, presenting the Museum Visitor Interactive Experience Model. This model explores visitors’ motivations, exhibition behaviors, post-visit responses, and social impact effects from three dimensions: personal context (interests, perceptions, knowledge, motivation, values, etc.), environmental context (architecture, exhibits, atmosphere, interpretive materials, etc.), and social context (peers, other visitors, museum staff, etc.) [35]. Subsequently, Falk published "The Museum Experience Revisited," using visitors’ "motivation to visit" as a starting point and focusing on the role of "identity" in the museum visitor experience. Falk criticized two prevailing perspectives in existing research on the museum visitor experience. The first perspective is institution-centered, suggesting that visitors’ reasons for visiting, the visiting process, and the construction of meaning all depend on the content and exhibits of the museum. The second perspective, although starting from the visitor, attempts to describe museum visitors solely through quantitative research methods based on demographic categories, visit frequencies, and social arrangements [36]. Falk argued that both of these research perspectives are limited, either based on simplistic stereotypes or relying on easily measurable indicators, and cannot comprehensively explain the deeper issues of the museum visitor experience. He chose to approach the study from the visitors’ perspective, conducting a type analysis based on the identity motive and delving into the roles of identity in pre-visit motivations, preset trajectories during visits, and post-visit satisfaction and memory [36]. In his exploration of the connection between identity and visitor learning, memory, and satisfaction, Falk’s research identified seven types of visitors and motivations based on identity: Explorers, Facilitators, Experience Seekers, Professional/Hobbyists, Rechargers, Respectful Pilgrims, and Affinity Seekers.

Falk’s relevant research was conducted against the backdrop of a decline in the number of museum visitors in the early 21st century in the United States, and was influenced by the theories and social context of North American leisure studies. As the definition of museums varies across countries, Falk particularly concentrated his investigation in the context of museum research in the United States. The study included not only art, history, children’s, and natural history museums, but also institutions like zoos, botanical gardens, and science centers. In contrast, China’s definition of museum domains does not necessarily include zoos, aquariums, or botanical gardens, but includes other types, such as red memorial museums and ethnic museums. This divergence makes the audience motivation types he proposed not universally applicable. Moreover, the widespread use of the internet, smartphones, social media, and changes in interpersonal communication may influence visitor motivations and types, prompting a continuous exploration of potential new scenarios. Simultaneously, it is essential to observe whether there are noteworthy differences in visitor types in Chinese museums compared to those abroad.

The research on museum visitor motivation in China commenced relatively late, and although it has gradually garnered attention in recent years, substantial research outcomes remain limited. According to Song Xiangguang, visitor motivations are primarily rooted in individual leisure values, lifestyle habits, and the specific needs associated with visiting particular museums and exhibitions [37]. Liu Yang’s study indicates that observing, peeping, and emotional needs are the primary motivations for visitors engaging in photography in museums [38]. Through questionnaire analysis, He Qijun and Gu Jing found that visitors’ motivations, such as pleasure, performance expectations, and effort expectations, exhibit strong explanatory power for their willingness to visit and behavior [39]. However, the aforementioned studies typically employed online questionnaires to investigate visitor motivations and behaviors, without distinguishing or delving into motivations based on different levels of museums and their audiences. This limitation provides the foundation and discussion space for the current study.

### Museum experience

The above-mentioned literature extensively reviewed the progress of museum visitor studies, exploring key conclusions and limitations in literature related to museum visitor types and motivations. While foreign researchers, exemplified by Falk, have conducted detailed and comprehensive studies on visitor motivations and behaviors, Chinese scholars have gradually recognized the significance of adopting a visitor-centric perspective in museum research. However, a critical issue remains. Theories and conclusions from foreign studies may not entirely represent or explain the specific circumstances in China. Moreover, due to significant differences between Chinese government policies on museum management, definitions, and classifications compared to foreign counterparts, and the influence of local culture and interpersonal communication patterns on visitors, there may be variations in audience behavior. Nevertheless, existing Chinese research has not thoroughly addressed these distinctions, nor has it adequately recognized their importance in cultural dissemination.

This study posits that museums, on the one hand, actively create diverse learning programs and environments for visitors. On the other hand, there is an increased emphasis on fostering interactivity, participation, emotional engagement, learning, and the development of experiential facilities. However, the effectiveness of these measures in meeting the diverse needs of visitors, further stimulating their active perception, imagination, understanding, and identification with the significance and cultural content of museum exhibits, as well as fostering national cultural confidence and cultural dissemination, has become a focal point in museum management and research. This issue involves the roles of museum visitor identities, exhibition perspectives, and experiential processes, accompanied by complex psychological and behavioral engagement in activities related to emotions, perceptions, and social interactions. In recent years, influenced by the societal background of the "experience economy" and related concepts and research, the assessment of "experiential value" has provided an essential research and measurement perspective for evaluating the perception and behavior of museum visitors.

The experience economy refers to the essence of customer consumption behavior, wherein customers spend money to engage in a series of non-material activities implemented according to their own preferences, or invest time in enjoying a sequence of memorable events provided by businesses [40]. In other words, "experience" emphasizes the incorporation of experiential details in the customer consumption process, rendering products or services more interactive, personalized, and considerate. This facilitates the comprehensive diffusion of the concept of the product, infecting the target audience in terms of both communication intensity and depth, establishing a more intuitive communicative interaction with the target audience [41]. In the measurement of experience, the fields of tourism and management often utilize the metric of experience value. Mathwick, Malhotra, & Rigdon (2001) posit that experience value is the customer’s perception and preference for the attributes or performance of a product, and the consumption of the product or the experience of the service itself also carries intrinsic value. This implies that individuals can derive experiential value from the actual use or distant appreciation of products and services [42]. Gallarza & Saura (2006) further confirm that experience value can directly influence customer experience satisfaction and subsequent behavior. Creating experiences that satisfy customers and evoke emotional perceptions is a key factor in gaining their acceptance and stimulating expected behaviors [43]. Specifically, customer endorsement of a particular product or service stems from the ability of that product or service to create value for them, and this value is a result of the interactive dynamics between the customer and the product or service. On the one hand, value involves the evaluation and comparison of two items or services, encompassing the benefits gained and the costs incurred during consumption with each individual’s assessment being subjective. On the other hand, value originates from the experiential aspects of the consumption process, rather than solely from the purchased product. Thus, value emerges in tandem with the overall experience [44]. In essence, the value customers derive from a product or service represents an elevation of the perceived service value or service quality. It manifests as a sense of spiritual satisfaction and gives rise to emotional, memory, and behavioral dimensions of identification. Additionally, the diverse experiential values that individuals derive from a product or service can influence their attitudes and actual behaviors toward that product or service. Furthermore, individual feedback on a product or service can impact the subsequent management of the product or service.

There is currently a considerable body of research on "museum experiences" that tends to focus on deconstructing visitors’ pre-visit expectations and on-site experiential dimensions. As illustrated by Sheng & Chen (2012), five types of visitor expectations were identified through a survey: relaxation and enjoyment, cultural entertainment, personal identity, historical reminiscence, and escapism from reality [45]. In other words, visitors’ motivations may stem from their relevant knowledge and professional background, interests, and the pursuit of specific information [46]. Participation in activities is equally crucial for enhancing the visitor experience [47]. Factors encountered by visitors before and after entering a museum, such as ticketing methods, reservation/queue times, transportation routes/convenience, indoor temperature/air quality, lighting, exhibition layout, exhibition area guidance, restroom location/quantity/quality, and the attitude/expertise of service personnel, can directly or indirectly impact visitors’ museum experiences. Jin et al. concluded that the museum experience is a process created collaboratively by multiple factors, including visitors, guides, and the environment [48]. Perceptions of museum content quality and emotional reactions are positively correlated with visitors’ satisfaction with their experience [49].

Based on these factors, this study posits that museum experiences encompass not only sensory aspects such as seeing, hearing, touching, and smelling, but also involve multiple psychological sensations such as communication and exploration. In other words, the visitor’s experience is influenced by the context, and their perspective is not confined to individual exhibits. Starting from the decision to visit, factors such as transportation, dining, companions, museum staff, and various situational aspects all contribute to shaping the overall experience [50]. Therefore, this study considers the museum as a comprehensive field for the generation of visitor experiences and utilizes experience value obtained by visitors before and after entering the museum as a metric to assess their sense of experience.

Bolton & Drew argue that using a single dimension to measure experience value is overly simplistic and suggest employing multiple dimensions for measurement [51]. Mathwick et al., summarizing past literature, identified four dimensions for measuring the experience value of products or services: service excellence, return on investment, aesthetics, and hedonics [42]. Additionally, referencing Rowley’s ten indicators for examining the quality of product or service experiences from a management perspective [52], this study categorizes six dimensions for measuring the value of museum experiences: (1) service quality (including ticketing/reservation channels, information inquiry channels, real-time exhibition activity information retrieval, and in-house service efficiency); (2) convenience (covering transportation, opening hours, and in-house public facility arrangements); (3) demographic positioning (considering inclusivity across age groups, social strata, and various demographics); (4) added value (beyond traditional exhibition and educational functions, encompassing leisure, mental and physical well-being, and social aspects); (5) interactivity (incorporating the use of digital technology); and (6) discount programs (including ticket prices, in-house creative products, and related goods or services). These dimensions are identified as focal points for research.

## Methods

This study adopts the perspectives of museum visitor patterns, motivations, and experience value, focusing on the Nan Song Deshou Palace Relics Site Museum in Hangzhou. Through case studies, participant observation, and in-depth interviews, the primary objective is to construct a profile of visitors to the museum in its current state. The aim is to provide empirical references for the museum’s subsequent service, marketing directions, and cultural communication. Additionally, the study intends to offer forward-looking and innovative strategic suggestions for Chinese museums to enhance service experience value and external communication.

Deshou Palace is located to the southwest of Lin’an Fu, the capital of the Nan Song Dynasty (present-day Hangzhou, Zhejiang Province). It stands out as one of the highest-ranking architectural structures in terms of ceremonial specifications during the Nan Song period. Traditional palace architecture and elements of garden aesthetics are widespread within, making it a masterpiece of Jiangnan gardens. It served as the residence for historical figures such as Emperor Gaozong, Empress Wu, Emperor Xiaozong, and Empress Xie. Over the past two decades, archaeological research has revealed that the Deshou Palace site covers an area of nearly 7000 square meters, including the foundations of large palaces, brick-paved roads, artificial hill foundations, drainage facilities, and various other relics. The protection and effective utilization of this site not only contribute to further exploration of contemporary values such as traditional architecture, Nan Song rituals, spiritual concepts, aesthetic life, and societal aspects, but also help enhance modern understanding and recognition of our country’s outstanding traditional culture. It plays a role in boosting internal cultural identity and external influence in the dissemination of Chinese culture.

The construction project of the Nan Song Deshou Palace Relics Site Museum in Hangzhou officially commenced in December 2020 and was formally opened to the public on November 19, 2022. Currently, the museum primarily operates on a reservation system, offering free admission to the public. Citizens and tourists can make advance reservations for visits through the official public account, with a booking window of one to three days. Additionally, individuals aged 70 and above may make on-site reservations by presenting their identification cards or senior citizen cards. The museum’s opening hours are from 9:30 AM to 5:00 PM every Tuesday through Sunday, with regular closure on Mondays.

This museum, leveraging archaeological remains, employs indoor exhibitions, digital artifacts, and simulated displays of archaeological sites to comprehensively present the historical and cultural panorama of the Nan Song Dynasty. After undergoing trial operations, observing the Spring Festival, and navigating through phases affected by pandemic-related policy adjustments, the daily visitor capacity of the museum increased from 500 to 1500 individuals. By the end of February 2023, the museum accommodated over 100,000 visitors, demonstrating the enthusiasm and engagement of residents in exploring the museum. Therefore, this study aims to use this museum as its focal point, seeking to uncover the current profile of visitors and their experiential perceptions. The findings will serve as a reference for the enhancement of service experience value and external communication for this museum and potentially other museums across the country.

This study employs an exploratory case study approach due to its status as a research method that involves "in-depth investigation of contemporary social phenomena based on real-life situations." This method not only facilitates the exploration of research questions, but also enhances the reliability and validity of research outcomes [53]. Data collection for the section on visitor experiences primarily adopts a "semi-structured, in-depth interview" approach. The initial interview outline is formulated based on the aforementioned literature and is further elaborated upon through in-depth questioning guided by the interviewee’s narratives. Formal interviews took place from February 10 to February 28 and from April 9 to April 21, 2023. Museum visitors were invited to participate in interviews through a "random sampling" method, and interviewee information was coded.

Prior to the formal interviews, the researcher conducted pre-tests with seven participants to assess the appropriateness of the interview outline. Following the pre-tests and subsequent revisions, the final interview content included: (1) personal basic information; (2) motivations for visiting the museum; (3) experiences during museum visits; (4) evaluation of museum facilities and services; (5) overall satisfaction with the museum; (6) willingness to communicate the museum experience; and (7) willingness to revisit the museum. The interviews lasted approximately one hour. As ethical review requirements and independent review units for studies using the in-depth interview method are not yet mandated in mainland China, the researcher, to ensure ethical legitimacy, informed participants of their identity, the purpose of the interview, and privacy protection measures before the pre-tests and the commencement of formal interviews. Consent for the use of participant information, interview data, and recordings was obtained orally or in writing. Ultimately, the study included thirty-four participants (14 males, 41%; 20 females, 59%), with ages primarily ranging from 19 to 45. Occupational distribution was diverse, and the majority of participants had been residing in Hangzhou or other cities and counties in Zhejiang Province for over two years.

In addition to the interview method, this study employed participatory observation and direct observation methods for data collection. This diversified approach is advantageous in overcoming the primary limitation of the case study method, which may lack generalizability in its conclusions [54]. Participatory observation was conducted concurrently with interview sessions, while direct observation was primarily concentrated in December 2022, January to April 2023, and other instances when the researcher visited the museum incidentally to obtain observational data. The purpose of direct observation was to collect information on the interactions of museum stakeholders, marketing activities, and their outcomes by recording observational insights, capturing on-site photographs, and documenting social interactions. These data serve as a supplement to the analysis and discussion sections of the interview results.

## Results and analysis

The data analysis in this study was conducted in two stages. In the first stage, which encompasses interview records, observation notes, on-site photographs, and descriptions, the raw data was coded based on visitor motivations and patterns. To enhance the objectivity of data processing and reduce errors, a "researcher triangulation verification" approach was employed. Two researchers were simultaneously involved in the data coding process to classify the current visitor patterns and motivational characteristics of the case museum.

In the second stage, building on the foundation of museum visitor experiences, perceptions, and behaviors, and referencing the concept of experience value and its measurement indicators, the interview data was aggregated and deeply analyzed. This aimed to synthesize and summarize the overall situations and issues related to museum visitor experiences, as well as the impact of these experiences on their perceptions and behaviors within the museum. By systematically analyzing museum construction, services, and existing cultural communication dynamics through these two perspectives, this study aimed to propose innovative paths and effective methods for continuous research and practical value in museum cultural communication.

### The motivations and profiles of the audience

The first stage comprised three steps. In the open coding phase, researchers (including investigators and two assisting coders) initially conducted a comprehensive analysis of all raw data. Subsequently, they integrated concepts that were identical or closely related, eliminated invalid concepts occurring less than twice, and focused on category formation based on the inherent relationships among concepts, ultimately resulting in the identification of thirty subcategories. In the axial coding phase, researchers analyzed and synthesized the thirty subcategories derived from open coding, consolidating relatively independent categories and distilling primary categories. This process revealed the interrelationships among different sections of interview data. Finally, selective coding was employed to refine core themes that were most pertinent to the research question and objectives, encompassing other related concepts. These core themes constituted the concepts and issues to be elaborated upon in subsequent research analysis, as outlined in Table 1.

**Table 1:** Interview data coding results.

Initial Concepts (with some content edited and condensed)	Secondary Categories	Primary Categories	Frequency of Occurrence	Core Categories
Interested in ancient Chinese culture, museums can help deepen cultural understanding and memory	Personal Interests	Memory Carriers	62	Knowledge Exploration
Museum visits provide a rich experience, with a habitual check-in	Experiential Habits			
Photo documentation aids in recalling the past	Life Records			
Visiting museums is meaningful, contributing to an understanding of history, society, and self	Self-reflection			
Appreciate cultural and artistic works from the Nan Song Dynasty, collecting professional materials	Data Collection	Professional Needs		
Examine the degree of restoration and authenticity of the Deshou Palace regarding Nan Song history, observe the staff’s skills in explaining Nan Song history	Knowledge Validation			
Reflect on the museum’s ability to disseminate Nan Song culture	Value Reflection			
Curious about the museum’s representation of Nan Song culture	Curiosity	Exploration Needs		
Guided from the former residence of Hu Xueyan to this point	Associative Consumption			
Explore potential collaborative opportunities with the museum	Business Opportunity Exploration			
Friends around me strongly recommended me to come here and take a look	Recommendations from Friends and Family	Social Conformity	55	Social Interaction
Afraid that a lack of common topics might lead to a breakdown in social relationships	Relationship Maintenance			
Impressed by the feedback on Deshou Palace from my classmates, which was praised by teachers	Peer Pressure			
I usually don’t have much to post on my social media, but I feel like visiting Deshou Palace is worth sharing	Social Discussion	Contextual Consumption		
Sharing relevant knowledge and visiting experiences with family, friends, and students	Experience Sharing			
The people I usually interact with are quite fixed, and visiting a museum provides an opportunity to meet new friends	Circle Expansion			
Visiting a museum with family can enhance communication time and strengthen relationships	Emotional Bonding			
The museum seems like a place that can isolate me from the world, helping me forget various unpleasant things in reality	Immersive Experiences	Escapism	41	Psychological Restoration
Traveling back to that era, I set an idealized life script	Self-Transformation			
If ancient emperors and officials lived in modern society, they would probably also be successful individuals	Role Perception	Energy Absorption		
Museums have a strong cultural infectiousness and soothing power, as if inspiring me to move forward with various historical anecdotes	Cultural Awareness			

#### Knowledge exploration

The desire for knowledge expresses itself in an interest in abstract thinking, complex thought processes, and philosophical discussions, as well as curiosity about various things [55]. Therefore, the desire for knowledge is often associated with museum visits [56]. As shown in Table 1, the "knowledge exploration" motivation/need was mentioned most frequently among the surveyed audience (62 occurrences). Furthermore, this study found that this motivation can be further subdivided into memory carriers, professional needs, and exploration needs. Firstly, visitors engaged in museum visits and photographing for the purpose of personal cultural knowledge and memory, interest, past experiences and perceived memories of museum visits [45,46], life records, and establishing memory cues [57], as well as self-reflection to re-understand society and oneself [45,57]. This is considered a space field for personal memory extraction, storage, and construction. In contrast to previous research, this study categorizes such needs as the memory-oriented, knowledge exploration type, mainly due to the audience’s invocation and construction of personal memory. Such needs were mostly mentioned by interviewees aged 35–50 and housewives.

Secondly, visitors may engage in museum visits due to their personal professional background or work requirements, aiming to collect relevant data, compare and contrast existing knowledge with real-world observations, identify problems, and contemplate solutions. Previous research has found that museums, as crucial institutions for informal learning, bear the responsibility of lifelong learning for the public. Museum visitors are generally considered individuals who prioritize learning, regardless of their identity, age, or profession, and learning is their primary motivation for visiting museums [58]. The results of this study further indicate that visitors going to museums for learning imply both active knowledge collection and argumentation and passive acquisition of work-related resources. For example, "I have been studying and appreciating artistic works from the Nan Song Dynasty, and I want to come to the museum to experience or test related knowledge. Perhaps it can provide inspiration for my research and creation" (Interviewee 21, female, 22 years old, college student, Hangzhou). Another example is, "To be honest, although I am not very accustomed to museums, which are relatively serious places, I work in this field and have to come here to take some materials" (Interviewee 13, female, 27 years old, self-media—travel blogger, Anhui).

Finally, visitors may engage in museum visits out of curiosity about attractions, from guidance from related sites, and to seek opportunities for resources and business. Research consistently confirms that curiosity is a vital intrinsic factor that triggers individuals to seek information and engage in behaviors [59–64], and similar results are found in studies related to museum visitor behavior [56,65,66]. Berlyne conceptualized curiosity as a response to novel stimuli, involving feelings of attractiveness and uncertainty [67]. Once individuals become curious about something, they experience anxiety, influencing their desire to acquire new knowledge and exploration strategies, with the goal of reducing their curiosity [62]. For instance, "I am curious about how Deshou Palace reproduces the architecture and palace furnishings of the Nan Song Dynasty…" (Interviewee 6, male, 30 years old, interior designer, Hangzhou).

This study also found that approximately 24% of the interviewees mentioned that they were attracted to visit Deshou Palace after visiting the "Hu Xueyan’s Former Residence" (located just across the street, less than a hundred meters away) due to the striking red exterior walls of Deshou Palace. Additionally, seeking "business opportunities" is an audience type identified in this study that distinguishes itself from previous research findings. Interviewees mentioning such needs include teachers, cultural and tourism workers, entrepreneurs, and business professionals. They believe that museums, especially newly established ones, may have untapped and potential spaces for development. Seizing these resources serves as a significant motivator for their visitation. This suggests that the motivation for museum visits is significantly influenced by personal professions or values. For example, "Looking for venues for organizing extracurricular activities and learning for students…" (Interviewee 3, male, 35 years old, middle school Chinese teacher, Hangzhou), and "Deshou Palace is part of a new cultural project, and there must be many resources that can be further developed and utilized, such as cultural and technological experiences…" (Interviewee 19, female, 31 years old, jewelry and cultural innovation entrepreneur, Beijing). In other words, driven by specific values and professional attitudes, visitors perceive museums as spaces necessary for exploring and obtaining personal work resources or capital. This also reflects the increasingly complex and diverse expectations and visitation demands of visitors to museums.

#### Social interaction

From the interview data, it can be inferred that this type of audience exhibits two types of motivational and behavioral states: passive social conformity and active contextual consumption. On the one hand, visitors engage in passive visitation behavior due to recommendations or persuasion from friends, fear of social relationship breakdown, and perception of peer capital. Moreover, these social factors to some extent create pressure for the audience to engage in "sharing and communication" and "capital comparison." The "social interaction" type of audience, as previously identified in domestic and international literature, tends to lean towards active willingness and behavior, lacking the capture and analysis of the motivation for visitors to museums based on "social pressure."

For example, "My mentor asked me to visit" (Interviewee 23, female, 31 years old, master’s student, Suzhou); "I’m not particularly interested in places like museums. This time in Hangzhou, a friend insisted on bringing me here to take a look" (Interviewee 17, female, 26 years old, film and television planner, Suzhou); "I saw Deshou Palace dominating my friends’ social media recently, and I heard it’s hard to make reservations. It seems to have become a hot topic. I feel like if I don’t come and experience it, I won’t be able to join in their conversations" (Interviewee 11, female, 19 years old, Hangzhou),; "I’ve never been here, so I can’t discuss it with my friends" (Interviewee 4, male, 39 years old, photographer, Hangzhou); "The comments on Deshou Palace made by my classmates were praised by the teachers" (Interviewee 11), "My classmate found a research direction here" (Interviewee 23); At the same time, visitors’ passive visitation behavior and perceived evaluations based on pressure will further influence their psychological and behavioral aspects of dissemination, for instance, "Avoiding self-exposure" (Interviewee 9, female, 60 years old, retired, Hangzhou; Interviewee 23); "Pressure on photography skills and retouching" (Interviewee 22, male, 23 years old, college student, Hangzhou); "No special feelings" (Interviewee 7, male, 24 years old, college student, Shanghai), all of which can affect the subsequent sharing and dissemination behavior of museum information by the audience.

On the other hand, visitors may also have an active willingness and behavior to visit museums for the purpose of establishing social topics, sharing experiences, breaking through existing circles, and enhancing relationships. Some interviewees indicated that visiting museums provides them with valuable social topics, such as "I usually don’t have much to post on my social media, but I feel like visiting Deshou Palace is worth sharing" (Interviewee 29, female, 32 years old, elementary school teacher, Ningbo). Knowledge and experiences gained from the museum can be shared with people around them, as mentioned by an interviewee: "Share the Nan Song culture and my visiting experiences with my students" (Interviewee 28, male, 38 years old, university teacher, Ningbo). Additionally, visiting museums can provide a space for establishing new social relationships, as stated by an interviewee: "Museums often organize various social activities such as lectures, workshops, exhibitions, etc., which gives me more opportunities to meet new friends and expand my social circle" (Interviewee 14, female, 34 years old, homemaker, Jinhua). It is worth noting that the establishment of such social relationships can indirectly bring positive impacts on their work efficiency and development, transforming the audience’s social interaction needs into knowledge exploration needs, such as seeking business opportunities." For example, "I think visiting museums is helpful for expanding interpersonal relationships. In the museum, I can interact and communicate with other visitors, make new friends, and the friends I meet here are likely to share similar interests with me. Like this Deshou Palace, I think people who come here should have some interest in Song culture like me. Perhaps there will be future collaboration in work" (Interviewee 19, female, 31 years old, jewelry and cultural innovation entrepreneur, Beijing).

In addition, previous research has confirmed that museums are places for "relaxation and fun" [45], "parent-child interaction" [68–70], and "relationship enhancement" [71], and this study also reached consistent conclusions. Approximately 32% of the interviewees visited with children, and they considered museums as places with cultural infectiousness (Interviewee 34, female, homemaker, Hangzhou), and educational significance (Interviewee 29; Interviewee 31, female, 60 years old, retired, Hangzhou; Interviewee 32, female, 58 years old, retired, Beijing). Museums provide space and opportunities for parent-child interaction (Interviewee 14, 34; Interviewee 25, female, 47 years old, freelancer, Suzhou), and contribute to enhancing mutual emotional communication (Interviewee 24, male, 37 years old, graphic designer, Hangzhou; Interviewee 29).

Another noteworthy phenomenon is that museum visitors driven by "friend recommendations," in addition to showing passive social conformity psychology, also exhibit some active adherence to social norms or behaviors. For example, "Seeing the photos recommended and shared by friends, it feels great, and I also want to come and take a look" (Interviewee 16, female, 34 years old, elementary school art teacher, Hangzhou); "I wanted to come here to take some photos to share on my social media, and I also want to buy some cultural and creative products to give to friends" (Interviewee 11). Along with their visiting behavior, environmental perception, and psychological changes, passive visitors may also transform into active contextual consumers, as stated by an interviewee: "Since I’m already here, buying some cultural and creative products as a souvenir or to give to friends is also a good idea" (Interviewee 23). This reflects that descriptions or photos about museums on social media, the acquisition and gifting of museum souvenirs, as well as discussions about museums in real life, are increasingly becoming media that influence the strength of social connections among audiences. Audiences are paying more attention to maintaining their personal social networks.

Additionally, this study found that social interaction is also influenced by the educational background and profession of museum visitors. This means that visitors with higher cultural knowledge reserves are more willing to share their visiting experiences with depth or personal unique insights with surrounding visitors or on social media. They may also exhibit motivations such as documenting personal experiences, using them for life experience reminiscence, and seeking recognition and interaction. For example, "I think visiting museums is a meaningful thing. I often share my experiences of visiting museums on Weibo. It not only helps me record my life trajectory and insights but also allows me to exchange ideas with friends and strangers through this sharing" (Interviewee 15, male, 58 years old, retired, Hangzhou). If engaged in education-related professions, they may visit and share experiences based on the factor of "interacting with students and promoting their knowledge improvement" (Interviewee 16, 28).

It is also important to note that, while satisfying their social interaction needs, audiences also engage in the exchange and enhancement of knowledge and culture during their social interactions. For example, "They have a really in-depth and comprehensive understanding of Nan Song culture. It feels like they came from that era. I really enjoy listening to them chat" (Interviewee 14). Another example is, "It’s amazing. I met my current business partner in the museum because we both have an interest in cultural relics, especially jewelry. Later, we discussed and learned related knowledge together and started our current jewelry and cultural innovation company. Because the jewelry we design incorporates some ancient totems, patterns, and other cultural elements, it is very popular in the foreign market. Especially in recent years, it can be seen that the love and interest in Chinese culture abroad have significantly increased. The cultural dissemination value and role of cultural and creative products are still significant" (Interviewee 19).

#### Psychological restoration

The concept of "psychological restoration" emphasizes the role of the museum as a space that separates from reality and provides an energizing environment. Firstly, audiences achieve the purpose of escaping reality and relieving stress through immersive experiences, imagining and reshaping self-image and identity. For example, "It feels like this is a place that can be isolated from the world, allowing me to forget all the bad things in reality…" (Interviewee 1, female, 21 years old, college student, Hangzhou). Escaping reality is one of the important expectations of visitors’ museum experiences [45]. Escaping reality can also stimulate visitors’ willingness to visit the museum [46]. Lee & Smith argue that the maintenance and utilization of the escaping reality function are essential for the marketing of historical sites and museums, because it allows visitors to escape the norms and problems of their daily lives, is more active, and emphasizes immersion more than passive experiential participation [72,73]. As expressed by interviewee 29, "Museums can temporarily make me forget about the pressures of reality, immersing myself in places that depict different eras and imagining the living scenes of ancient people." The attention, sensory changes, and emotional shifts that visitors experience in museums, even reaching a state transcending reality, are referred to by Hirschman as "aesthetic experiences" [74]. From the perspective of the experience economy, aesthetic experiences are a combination of passive participation and immersion [73]. In other words, even if the original motivation for museum visits is not explicitly seeking psychological restoration, visitors, when engaging in the visitation behavior, also obtain varying degrees of aesthetic experiences closely related to their sense of immersion.

Furthermore, visitors may absorb positive energy by perceiving the personalities, abilities of historical figures, and the cultural forces. According to Petkus, visitors can create a "new reality or role" for themselves in museums [75]. For example, "Museums can give me a kind of time-traveling experience, allowing me to imagine ancient times, reflect on the modern era, and set my preferred life script…" (Interviewee 5, female, 34 years old, master’s student, Shanghai). Additionally, sentiments like "I think ancient emperors and officials, if they lived in modern society, would also be successful individuals…" (Interviewee 1), and "Museums have a strong cultural infectivity and comforting power, as if inspiring me to move forward with various historical anecdotes" (Interviewee 10, female, 38 years old, university teacher, Hangzhou; Interviewee 19), underscore the audience’s focus on self and inner world. They aim to achieve self-healing and elevate their life values by perceiving successful figures and cultures from the past. It is important to note that, while museums have the potential to restore the psychology or negative emotions of visitors, this potential is effective only for those who have already had a positive experience in the museum.

In this interview, it was emphasized that the motivation for "psychological restoration" was more prominent among female participants, accounting for 35% of all interviewees, and on average, they mentioned this motivation one to three times. In contrast, male participants who mentioned this motivation constituted only 8% of all interviewees. This phenomenon largely indicates that, in addition to being influenced by their motivations for knowledge exploration and social interaction, women are more concerned about the psychological perception and restoration aspects of museum visits compared to men. The reason may be that, compared to women, men typically exhibit a more rational approach to problem-solving and stress/emotion management. Regarding museums, especially in institutions like the Nan Song Deshou Palace Relics Site Museum, men may not perceive them as places or means to relieve stress, let alone visit them for the purpose of psychological restoration.

Finally, about 85% of the interviewees, when describing their museum visiting experiences, motivations and expectations, evaluations, overall satisfaction, and subsequent behaviors, expressed higher expectations and requirements for the overall environment and space creation of the museum. Among these aspects, "atmosphere" and "immersion" were the most frequently mentioned keywords during the interview process. These two keywords encompass various elements within the museum, such as the number of visitors, background music, use of technology, lighting, leisure settings, and more. They significantly influence the overall attitude (satisfaction) and behavioral intentions of museum visitors (such as revisiting and recommending), making them crucial aspects for further discussion in this study.

### The assessment of the experiential value of museums

Drawing on the concept of "experiential value" from the field of marketing management and examining the indicators of product or service experiential quality proposed by Rowley (1999) [52], Table 2, in conjunction with the practical insights from interviews in this study, assesses the experiential value of the current case museum from six aspects: service quality, convenience, demographic positioning, added value, interactivity, and promotional schemes. It is important to note that, as this section primarily focuses on the assessment of museum experiential value, some overlap may exist in the oral data from the interviewees. Therefore, individual participant identifiers are omitted, and the researcher has moderately summarized and consolidated the information while preserving the original intent expressed by the participants.

**Table 2:** Visitors’ perspectives of approving the museum experience value.

Indicators	Brief description of the concept	Summary of interviewees’ experiences
Quality of Service	Efficiency and quality of all museum services received by visitors	a. We provide tour guide equipment rental, code scanning tour, and reservation for human interpretation service. b. Limited reservation access, and difficulty to get a suitable date and time c. The time limit for entering the museum is too strict, and the security guards are not very efficient in guiding and verifying the reservation information. d. There are more visitors with children in the museum during holidays, and the exhibition line is disorganized, which affects the visiting experience. e. Need to improve the museum guide system f. Fewer leisure seats in the museum g. disabled access and service system is not complete h. The manual interpretation service of cultural relics in the museum is to the textbook, which will reduce the patience and interest in learning and prefer the storytelling and humorous interpretation of foreign guides. i. Too little historical content and form of explanation of Nan Song culture
Convenience	Accessibility of museum-related information and services	a.Visiting the former residence of Hu Xueyan will bring you to Deshou Palace by the way b. The traffic is not convenient, and there are no obvious signs at the subway entrance, intersection, bus station, etc. c. The publicity of the museum is not effective, and people are not informed of the contents and activities of the museum in a timely and accurate manner. d. There is no paper floor plan or brochure in the museum, which may reduce the willingness to promote the museum to friends and relatives.
Demographics	Reasonableness or relevance of the museum’s customer orientation to the actual age structure of the customer, and the corresponding service content settings	a. Most of the visitors are tourists, most of them just go to take pictures, especially the red wall of the museum b. The history, architectural aesthetics, and cultural value of museums are important factors that attract professionals to visit c. Museums are a way to pass the time and enhance emotions, so they invite friends and family to visit with them. d.Government-related institutions and business-related organizations will arrange the visit
Added Value	In addition to the traditional display and educational functions and values, the setting and quality of other services and functions such as leisure, healing, socialization, etc.	a. The museum provides some creative drinks, food and goods, but the quality needs to be improved. b. There is no cafe or teahouse in or around the museum, which will reduce the willingness to stop to learn, promote and revisit c. The museum visit experience is the most important, and they will not consume creative goods. d. The creative development of museums is an important element in evaluating their quality e. The architecture and overall space design of Deshou Palace will make people feel relaxed, revering the wisdom of the ancients and the imagination of traveling to ancient times f. The museum can plan regular lecture activities, which will increase the willingness of visitors to come and promote the museum. g.The film screening room should be equipped with additional leisure seats
Interactivity	The extent to which the museum uses technology and how it uses multimedia resources to interact with visitors and to stimulate and meet their needs for information, entertainment and participation at the same time	a. The operating system and content of the smart guide service are not convenient and simple enough to receive. b.The use of the personal tour equipment provided by the museum can avoid the problem of walking around and crowding in group tours. c.The technology display of the Nan Song Deshou palace relics site site is very attractive d. The botanical landscape is attractive and often considered part of the exhibition, but most are simulated plants, which can reduce their quality satisfaction e. It will be more touching after checking and understanding the relevant historical and cultural information on the Internet in advance f. The lack of interactive service experience, play, and learning programs in the pavilion will reduce the interest in youth participation and learning, as well as children’s willingness to visit
Preferential Program	Ticket prices or other associated consumer discounts	a.At present, the museum adopts the reservation system and waives the admission fee, although it will increase the willingness of some people to visit. b. Based on the quality consideration, we do not reject the admission fee or service fee of the museum. c. Hope to add special exhibitions or lecture activities d. We hope to provide discounts and services related to the museum’s cultural and creative consumption and transportation or other tourist attractions (such as the former residence of Hu Xueyan).

From Table 2, it can be observed that, when examined from the perspective of museum visitors, the experiential value of the current case museum exhibits the following issues and characteristics:

#### Museum circulation and layout, staff services, and communication satisfaction

Contemporary museum visitors’ evaluations of museum satisfaction have transcended the historical dimension primarily focused on "objects" (collection, exhibition, research, etc.). Instead, there is now an emphasis on achieving self-service experiences and spatial experiential sensations.

The "layout and circulation" of the museum, including guidance and smoothness, are crucial factors influencing visitor experience and their evaluation, revisit intention, and willingness to disseminate. Some visitors expressed challenges related to their museum experience: "I cannot determine my visiting route in the museum" (Participants 6, 23, 27); "I prefer to grab a flat map upon entering the museum, allowing me to efficiently tour the entire museum and serve as a souvenir" (Participants 12, 15, 29); "Upon entering the exhibition area, I hesitate about whether to go left or right" (Participants 14, 17, 33); "I think the visiting route and distribution of exhibition areas inside are not reasonable. Without accidentally encountering a guide, I thought my visit was over" (Participants 3, 30, 31); "I still need to personally explore it before bringing friends" (Participants 10, 28); "I think I will share on my social media how I got lost in this museum" (Participants 11, 25).

Museum visitors have higher expectations for curator services, including entrance guidance and traffic control, as well as collection explanations. Their impressions of the museum and willingness to disseminate information are closely related to these services. Some expressed concerns: "I feel the entrance control is too strict; you must enter exactly at the scheduled time, not even a minute early. However, there are clearly some places outside the museum where you can take photos, and there aren’t many people. It wouldn’t affect the museum environment or others" (Participants 13, 22); "It was raining outside today, and I wanted to enter a few minutes early because there was basically no place to shelter from the rain nearby. The security guard at the entrance kept refusing, and the attitude was particularly bad. I haven’t encountered this situation when visiting other museums" (Participants 9, 26). Some participants suggested that the museum should expand reservation channels and daily visitor quotas, because "it’s challenging to make a reservation" (Participants 4, 12, 15, 24), allowing more people to visit the museum more conveniently and pleasantly. In addition, some participants mentioned issues with the rented audio guide devices (Participant 3) and found that manual explanations lacked interest or storytelling elements (Participants 8, 9, 17, 32), suggesting that they were not as engaging as external tour guides (Participants 11, 12, 17, 19).

The museum’s external promotion efforts and the forms and effects of cultural dissemination also serve as important indicators for assessing the museum’s service quality. Some participants expressed their opinions on the museum’s promotion: "I usually only see Deshou Palace’s promotion on my friends’ timeline. I think its promotional efforts are not enough" (Participants 10, 22); "Deshou Palace should do more promotion on the learning aspects of history, culture, or architectural aesthetics in the media, or add some related settings, which should be more attractive" (Participants 5, 33). Some participants also mentioned challenges like "the museum entrance is hard to find" (Participants 4, 11), "lack of guidance on transportation routes" (Participants 1, 2, 4, 8–15, 18, 20–22), and "seemingly no specific cultural discussions or lectures during certain periods like Today Art Museum." At the same time, the participants’ dissemination behaviors and purposes are closely related to the museum’s external promotion strategies and forms of cultural dissemination. Sixty-five percent of the participants stated that the short videos or promotional images related to the museum on the internet mostly focus on the red walls, architecture, and limited cultural and creative products. The cultural uniqueness and the unique value of Nan Song culture are not fully and effectively communicated. Therefore, visitors’ external dissemination forms, such as taking photos and checking in, tend to focus more on recording and social interaction, rather than cultural explanations and dissemination. For example, one participant said, "I often see everyone taking pictures with the red walls on the internet, feeling retro and beautiful. Why should I spread it? It’s just to let others see that I also have a set of beautiful photos, spreading Song culture… I don’t have any thoughts. I feel like I haven’t seen anything special, or maybe I don’t understand" (Participant 18). Another participant mentioned, "If you want to understand the unique value and cultural aspects of the museum, you still need to search for relevant textual introductions online" (Participants 3, 4, 15, 21, 31).

##### Cultural and creative consumption

Utilizing traditional culture and cultural heritage resources to develop cultural and creative products represents a significant collaboration between museums and businesses. This collaboration serves as a crucial avenue for cultural dissemination and constitutes a vital operational strategy for enhancing museum revenue and ensuring sustainable development. Moreover, the growing demands of contemporary society for spiritual, cultural, and aesthetic consumption play a pivotal role in promoting the development of cultural values within museums. However, the research conducted revealed substantial disparities in the consumption intentions and attitudes towards museum cultural and creative products among museum visitors.

Firstly, the everyday consumption habits of the general public significantly influence their behavior. Some interviewees reported a lack of prior consumption habits related to cultural and creative products, stating sentiments such as "Cultural learning in museums and the immersive experience are more appealing to me" (Interviewee 3), and "The importance lies in the experience and memories" (Interviewee 9). On the other hand, some participants expressed a different perspective with statements like, "Every time I visit a museum, I buy some cultural and creative products as souvenirs."

Secondly, the value, price, and practicality of cultural and creative products emerge as crucial considerations influencing the purchasing behavior of museum visitors. For instance, sentiments such as "Authentic artifacts are more valuable than replicas or designed ones" (Interviewee 16); "It looks great, but it’s not very useful to buy" (Interviewee 7); and "Not practical and too expensive" (Interviewee 12, 23) were expressed. Additionally, the social communication and interactive functions of museum cultural and creative products can contribute to the potential allure and promotional value of museums. Statements like "Cultural and creative products make artifacts more warm and evoke a sense of historical familiarity" (Interviewee 19); "My friend loves collecting museum cultural and creative products, so I always pick one for her each time I visit" (Interviewee 18); and "A friend has one, and I found it very attractive, so I came to buy one" (Interviewee 21), highlight the social and interactive dimensions of museum cultural and creative offerings.

Thirdly, the "originality" of cultural and creative products emerges as a primary factor stimulating consumer willingness to purchase. Statements such as "Cultural and creative products from each museum are unique" (Interviewee 21); "I mainly buy it to give to friends; it feels unique and shows that I have carefully selected the gift" (Interviewee 2); and "I won’t buy the same thing as others, or basically won’t present the same thing when giving gifts" (Interviewee 17), highlight the appeal of uniqueness. However, some interviewees also noted that similar or identical products are available on online platforms like Taobao (Interviewee 11, 25).

##### Holistic cultural learning and interactive experience

Goode (1891) argued that museums should not merely serve as repositories for artifacts, but rather become sources of intellectual stimulation and new ideas [14]. As a medium for cultural exchange with the public, museums should possess characteristics and values beyond their traditional functions of collecting and displaying artifacts. They should also serve as platforms for expressing identity, expanding intellectual dimensions, facilitating public education, and promoting cultural communication both domestically and internationally. For visitors to museums, the aesthetic layout, level of interest, and the cultural or learning atmosphere and experience created by the movement of different strata within the museum space are crucial factors influencing their perception, evaluation, and behavior. This includes aspects such as revisiting museums, external communication, and changes in daily behavioral habits.

From the interview data, it is evident that the current audience profile of the museum is primarily composed of individuals in the 20 to 40 age group. The occupational distribution is quite diverse, with the predominant types of visits being for sightseeing, tourism, and group educational tours. Museum visitors, drawing on their individual characteristics such as motivations, interests, habits, experiences, and values, engage in a range of cultural practices involving interactions between individuals and between individuals and objects within the museum. During these experiences, the spatial layout and supporting facilities within the museum are observed, experienced, and evaluated in the context of tourism landscapes, cultural elements, and leisure and entertainment domains.

Most visitors take photographs for various purposes, including commemoration, learning, and social interaction. Quotes from interviewees such as "This will become a part of my life memories" (Interviewee 12); "It can be used as educational material" (Interviewee 6); and "It will be an artistic addition to my social media" (Interviewee 16), reflect the diverse motivations behind capturing images. However, the current service facilities in museums have not adequately addressed the needs of the entire audience, posing obstacles to the immersive experience of artifact displays, visitor engagement, and cultural absorption within the museum space.

Many interviewees expressed concerns about the presence of noisy children (Interviewees 2, 3, 5, 11, 17, 20) and the lack of rest areas (Interviewees 7, 9, 12, 13, 15). In response, museums should consider the learning characteristics and behaviors of children. They could introduce a "Children’s Leisure Area" (Interviewee 10), or an "Interactive Learning Zone" (Interviewees 20, 33), to mitigate disturbances to other visitors’ experiences. Additionally, attention should be given to the needs of parents with young children, the elderly, and individuals with mobility challenges. This may involve the provision of leisure seating, baby stroller amenities, nursing rooms, wheelchairs, and the establishment of volunteer service stations within the museum to enhance visitor satisfaction with the exhibition and service experience.

For visitors with a certain level of expertise, the main requirements during their museum visit include learning, clarification, discussion, and the opportunity to refresh their knowledge. Quotes from interviewees such as "I would be more attracted if there were lectures or discussions specifically focusing on the history and artifacts of the Nan Song Deshou Palace Relics Site Museum" (Interviewee 9); "The explanations of cultural relics are too textbook-like" (Interviewees 10, 24); "There is a lack of interactive learning elements" (Interviewee 5); and "It would be better if there were virtual exhibitions or immersive explanations online" (Interviewee 13), emphasize the desire for more in-depth engagement. These visitors exhibit a strong curiosity and eagerness to acquire knowledge, especially in areas where their understanding is limited or unclear. They actively seek explanatory services, as reflected in statements such as "I usually join guided tours booked by groups and sometimes ask questions" (Interviewees 9; 31); "Some information is not available online" (Interviewee 13); and "There are many unclear points that I want to get answers to promptly" (Interviewee 11). Additionally, interviewees mention seeking clarification from others and engaging in discussions, such as "I will try to ask people around me for advice or discuss" (Interviewee 8).

In addition, unconstrained exploration, immersive imagination, interaction, and self-healing are often the experiences and cultural learning expectations of female interviewees and older individuals. For these visitors, elements such as museum space design, artifacts, lighting, and even scents contribute to and stimulate their imaginative experiences. Quotes like "I like to walk quietly in the museum, looking at the dormant yet vivid artifacts; it’s like entering another world" (Interviewee 18); "Travelling through history, I transport myself back to ancient times, as if engaging in a dialogue across time and space with them; it feels great" (Interviewee 15); and "The combination of architecture, plants, lighting, display cases, and even scents is very comforting and therapeutic," highlight the importance of these elements in creating a profound museum experience. However, the concern of being disturbed by others (Interviewee 10) emerges as a significant obstacle to the immersive experience of such visitors. Simultaneously, some interviewees express a desire for technology-assisted immersive experiences and learning: "I would hope that this museum could have some VR or AR experience projects that allow me to better understand the social and cultural landscape of that time" (Interviewee 19).

##### Consumption demands with interrelated needs

The contemporary public’s perception of museums has transcended the role of a purely cultural and educational institution. It is now evaluated as a comprehensive public service entity that encompasses leisure and entertainment, social interaction, cultural dissemination, cultural innovation, technology transfer, and educational tourism services [76]. Among these aspects, 70% of visitors mentioned "experiential satisfaction" and "convenience." According to research data, the current museum audience primarily consists of the younger generation from the later stages of China’s reform and opening-up period. Their emotional and lifestyle demands not only surpass those of their predecessors, but also place a greater emphasis on enjoyment, experience, and innovation. This shift in artistic and cultural consumption has become a prominent characteristic of the contemporary museum audience.

Currently, the museum operates on a reservation system and offers free admission, aiming to showcase the Chinese Nan Song rituals and aesthetic values to the general public. This approach creates a more open environment for cultural education and dissemination. While this strategy is beneficial for attracting visitors, it poses challenges in controlling the museum’s service quality and overall experiential atmosphere. Some interviewees expressed a willingness to pay an entrance fee, stating, "I don’t mind paying for admission" (Interviewees 7, 27–29) and "I would rather spend money if it means more investment in service facilities." The museum’s service facilities include "creative dining" (Interviewees 13, 15), "cultural books" (Interviewees 1, 5), "cultural and creative products" (Interviewees 2, 17, 18, 19, 21, 29), "lectures" (Interviewees 9, 33), and "special exhibitions" (Interviewees 11, 31–33).

Additionally, convenience is a crucial factor influencing visitors’ willingness to explore and their overall satisfaction, impacting the practical experience of museum-goers. Interviewees express concerns about the convenience of visiting with statements like "I pay attention to the convenience of transportation to the museum" (Interviewees 7, 11), and "I didn’t see clear signs directing to the museum when I exited the subway station today" (Interviewees 9, 23). Furthermore, convenience extends to cultural consumption and the interaction of leisure, sightseeing, and entertainment within the museum’s surroundings. Comments such as "I noticed this museum after leaving Hu Xueyan’s former residence" (Interviewees 19, 31); "It would be great if there were combo tickets" (Interviewees 21, 28); and "It’s inconvenient to find food nearby, and there are no distinctive restaurants or cafes; it gets boring when queuing" (Interviewee 11), highlight the multifaceted nature of convenience in the museum experience.

### Conclusion and discussion

This study focuses on the typology, motivations, and experiential values of museum visitors, using the Nan Song Deshou Palace Relics Site Museum in Hangzhou as the research subject. Data were gathered through case studies, participant observations, and in-depth interviews. Building upon the classifications of museum visitors identified by scholars domestically and internationally, the study referenced the concept of experiential value from the business domain and employed related scales as evaluation criteria for assessing the quality of museum services and communication. The primary objective was to unveil the diverse profiles of visitors to the Nan Song Deshou Palace Relics Site Museum in Hangzhou at its current stage. The study aimed to explore the experiences of visitors and cultural communication strategies employed by the museum. Ultimately, the research seeks to provide practical insights for Chinese museums in examining and refining their service and communication systems.

### The characteristics of museum visitor profiles

At the current stage, the visiting patterns of the audience primarily manifest as "knowledge exploration," "social interaction," and "psychological restoration." While similar classifications or conclusions have been drawn in previous domestic and international research, this study, focusing on the Chinese context and unique research samples, still yields some distinctive results that differ from previous findings.

Firstly, the current visitors to the museum are primarily motivated by "knowledge exploration," encompassing: (1) "memory-oriented" exploration involving experiences of recalling and constructing memories, emphasizing the extraction, construction, and storage of personal memories; (2) "professional-oriented" knowledge gathering involving knowledge collection, argumentation, and resource acquisition, demonstrating both proactive and passive behavioral tendencies; (3) "curiosity-oriented" exploration encompassing actions related to location, knowledge, and resources/capital exploration with a highlighted influence of "location association." This also reflects a compensation for personal psychological uncertainty to some extent. In comparison to more traditional forms of cultural learning, the museum, as an informal learning environment with diversity and flexibility in terms of objects, content, and time, serves as a voluntary and actively guided lifelong learning mechanism [77,78]. It leverages factors such as audience interests, curiosity, exploration, application, imagination, task achievement, and community interaction to positively impact knowledge and skill enhancement, stimulate creativity, and facilitate attitude and behavioral changes. The museum can be seen as an important avenue for voluntary and actively guided lifelong learning and a crucial pathway for cultural dissemination.

Secondly, visitors’ exploration behavior is stimulated by the need for "social interaction," resulting in two behavioral states, more passive social conformity and active domain consumption. This study reveals that visitors demonstrate both active and passive social conformity tendencies when influenced by "friend recommendations." Simultaneously, they may experience pressure to "share and communicate" and engage in "capital comparison" due to fear of social relationship disruption or perceived peer or learning capital acquisition. This pressure further stimulates their willingness and behavior to passively visit the museum. Active visitors exhibit more positive and enjoyable experiences, while passive visitors tend to be more negative and complete the museum visit task in a more "checklist" style. Furthermore, visitors may actively engage in museum visits to fulfill their needs for establishing social topics, sharing experiences, breaking through existing circles, and enhancing relationships. This study suggests that visitors who establish new social relationships within the museum domain may indirectly contribute to the improvement and development of their work efficacy, transforming their "social interaction" needs into "knowledge exploration" needs, such as "seeking business opportunities." This highlights the museum’s contemporary role as a "public forum," as described by Cameron [79]. The attributes and value of its social media are evident, and its ability to gain group belonging and identity on public spaces and online platforms becomes a crucial factor influencing visitor behavior [12]. Museums create possibilities for local cultural heritage and communication as well as cross-cultural exchange [10–12]. Consequently, relevant research should move beyond the discussion of museum functions and place more emphasis on understanding the impact of audience psychology and behavior on museum construction and cultural communication outcomes. It is worth noting that visitors strongly influenced by social factors may have potential value in disseminating the museum’s image and culture. However, understanding the factors that (dis)satisfy their social needs and exploring the emotional and behavioral consequences after (dis)satisfaction remain directions for further investigation.

Thirdly, visitors are increasingly demonstrating a motivation for "psychological restoration" and expectations for the corresponding experiences. This motivation includes not only the recognition and expectation of the museum as a space for escaping reality and relieving stress, but also the perception and imagination of the museum providing energy. In this study, female visitors emphasized this motivational aspect and the acquisition of psychological and spatial resources more than male visitors. This type of visitor typically has higher expectations and demands for the atmosphere, space, and environmental settings of the museum. Although their motivations for exploration, learning, and social interaction may be relatively weaker, they often engage in a more sensitive and detailed exploration and observation of museum space planning, exhibitions, and other visitor behaviors. This makes them valuable for improving and enhancing the quality of museum services and the effectiveness of cultural communication.

These research results indicate that the motivations of "knowledge exploration," "social interaction," and "psychological restoration" are not isolated within individual visitors’ intentions and behaviors. Instead, they dynamically change along with visitors’ museum experiences and environmental perceptions. Drawing on Falk and Dierking’s (2016) developed interactive experience model, visitors’ museum experiences can be decomposed into three dimensions: personal context, social context, and physical context [36]. In line with the results of this study, this division can be described as follows: (1) personal context, encompassing the collection of visitors’ individual characteristics (including genetics), incorporating their prior knowledge, experiences, interests, and tendencies in selecting and utilizing museum resources; (2) social context reflecting visitors’ preferences for social interaction behavior, such as perceptions, attitudes, and communication willingness towards companions, venue guides, interpreters, etc.; and (3) physical context associated with museum space, lighting, temperature, information boards, exhibits, service facilities, etc., potentially impacting visitors’ immersive experiences and satisfaction. These three dimensions work in conjunction to help visitors understand and experience museum spaces and exhibits [80]. They collectively influence visitors’ subsequent behaviors.

Overall, the factors influencing the cultural perception and behaviors of modern museum visitors are complex and diverse [2]. Analyzing museum visitor types can provide insights into grasping the diverse profiles of modern museum visitors, guiding the adjustment and formulation of more precise museum management and communication strategies. Jin & Zhang propose that museums should be places for free learning, immersive spaces that inspire creativity and free imagination, havens for safe, quiet, independent thinking, and healing spaces that help visitors relieve stress and regulate emotions [81]. The results of this study also indicate that the historical and cultural value, architectural aesthetics, spatial functionality, and social attributes of museums are key motivators for public visiting experiences. Therefore, museums need to carefully consider and improve the following aspects of their spatial functionalities and continuous maintenance systems: free learning, free imagination, deep immersion, independent thinking, social interaction, psychological healing, and emotion regulation. This will meet the expectations and diverse needs of different types of visitors, ultimately enhancing their satisfaction with the museum and fostering cultural understanding, identity, and widespread dissemination.

### The value of museum experience

From the perspective of the current quality of museum experiences, there are several main issues and characteristics. Four issues lie in suboptimal circulation and layout, inadequate curator services, and dissatisfaction with promotion and communication. Additionally, there is a need for improvement in cultural learning and interactive experience programs for the entire audience. The characteristics manifest as a differentiation in museum cultural and creative consumption, and the emergence of associated consumption demands. In terms of the issues, museums, as spaces for disseminating knowledge and culture and providing visitors with experiential opportunities [4], are intricately linked to visitors’ perceptions and behaviors related to learning, imagination, self-realization, and interaction. This includes the smoothness of circulation, the rationality and connectivity of structural layout, the complementarity of functional project offerings, the creation of a conducive atmosphere, and the quality of both internal and external personnel services. If museum visitors have a subpar experience, it is likely to result in physical or mental fatigue during the exhibition process [82], subsequently reducing their willingness to linger, revisit, and disseminate. Simultaneously, different types of museum visitors demonstrate the practical significance of knowledge construction, self-construction, and the establishment and maintenance of social networks during their exhibition experience.

According to previous research findings, the public’s purposes for museum visits include fulfilling dimensions, such as leisure and entertainment, relaxation, self-identity construction, memory exploration and creation, and escaping real-life pressures [45]. Individuals with high curiosity and professionals/enthusiasts generally expect diverse cultural learning experiences, those with social needs anticipate better interaction spaces and conditions, and individuals with a need for psychological healing require better immersion and freedom to complete self-identity construction and meaning-making. Research data indicate that if visitors can harvest positive emotions and physical experiences during their exhibition process, their museum satisfaction, practical happiness, and dissemination willingness will significantly increase. This viewpoint aligns with the results of Jin & Zhang’s study, suggesting a positive relationship between the process of meaning construction by museum visitors and their sense of happiness [81]. In other words, the "meaning-imbuing" capacity created by museum spaces and artifacts can interact and jointly influence visitors’ experiences, further impacting museum development and its cultural communication effectiveness.

This study also discovered that, in addition to the museum’s original functions of exhibition, collection, education, cultural communication, as well as the leisure, entertainment, and social interaction functions bestowed by the modern era, different types of visitors, influenced by their identity and social networks during the exhibition process, increasingly demonstrate characteristics of self-identity acquisition and reconstruction. This is an aspect that has been rarely explored and demonstrated in previous research. For instance, individuals with a teacher identity may focus on the perspective and methods of knowledge dialogue and dissemination, and those with parental roles may lean towards an educational perspective, paying attention to the accessibility and fun of cultural explanations. On the other hand, professionals (in cultural studies, architecture, photography) may emphasize innovation and knowledge recombination, often reflecting a "social" need. As per Wilson & Harris’s perspective [83], visitors intend to establish and maintain social networks and gain social recognition during their museum visits, thereby defining and empowering their self-identity.

Looking at the characteristics, museum visitors show a demand for cultural and related consumption. In the early days, museums were centered around objects, emphasizing the collection and display of artifacts as well as the educational function of culture [4]. However, with changes in society, the consumer market, and public perceptions, more attention has been given to the cultural communication value and forms of museums. Obtaining perception and experience in the "visual" space and triggering action are important aspects to consider when realizing the regional and national cultural identity and external communication needs. The combination of objects within the museum collectively forms the cultural narrative in the museum space [15,22]. Therefore, in exploring the cultural communication path of museums, it is necessary to simultaneously consider and study the spiritual and symbolic value of objects in shaping and disseminating culture. Using various means such as visuality, quality, innovation, technology, and interactivity to invite visitors to experience and learn about culture [84]. However, current domestic museums face issues in the conceptual lag, low enthusiasm for funding input, lack of creativity and innovation, insufficient distinctiveness, simple labeling, and excessive commercialization in the development of the spiritual and symbolic values of objects [85], leading to a differentiated landscape in visitor consumption of museum cultural and creative products. Based on the empirical research results of Song & Feng (2020) on consumers of cultural and creative products nationwide, the current characteristics of public consumption of museum cultural and creative products are as follows: consumers prefer light cultural and creative products such as food, accessories, and stationery; consumers pay more attention to the "beauty, fun, and quality" of cultural and creative products, and "low price" does not necessarily drive purchasing power; 300 yuan is the price threshold, and high creative added value is expected to promote consumption upgrading; the overall experience in physical stores is good, but there are shortcomings in product display and service; and online and offline channels each have their own strengths, fully tapping into urban spatial possibilities, and expanding cultural and creative sales channels. These survey results are partially confirmed in this study. This clearly indicates that museums and related practitioners can only meet the objectives of supply and precise communication by developing and designing cultural and creative products from the perspective of consumer demand, while also improving service and experience quality.

Finally, museum construction and communication constitute a multidimensional systematic project involving the design of the public, culture, marketing, and more. Viewed from the perspective of "people’s" perceptual experience and behavior, this involves collaboration between the government and various sectors of society. Taking this study as an example, museum visitors expressed a strong demand for convenience and associative consumption, such as the combination, sale, and promotion of transportation, dining, and entertainment options. Ticket revenue is no longer the focus of attention for operators and consumers. Instead, the distribution of cultural and creative products, books, dining options within the museum, and shopping, leisure, and entertainment formats outside the museum are key elements for comprehensively enhancing museum revenue and cultural communication effectiveness.

### Museum construction and communication strategies

Based on the research results, this study proposes the following recommendations for museum construction and communication:

1. Emphasize the improvement of the internal layout fluency and the comprehensive nature of service facilities. Continuously enhance and upgrade service quality while prioritizing the development of an online and offline cultural communication system and knowledge platform. Establish a digital exhibition and cultural heritage preservation center [86], utilizing modern technological means for digital preservation, display, and dissemination of Nan Song culture. Provide the public with a more comprehensive and diverse visiting experience, thereby extending the time and space for exhibition and learning. This approach aims to stimulate the public’s willingness and actual behavior in dissemination, ultimately expanding the museum’s communicative influence.
2. Beyond showcasing artifacts, museums should incorporate highly interactive and participatory display methods, such as virtual reality and augmented reality experiential interactive projects. This immersive approach facilitates the output of cultural content and allows for the organization of lectures, explanations, and research activities to attract scholars, experts, and enthusiasts, enhancing the educational and research value of the museum. Collaboration with schools and communities can further amplify the benefits, comprehensively improving the effectiveness of public cultural learning and communication.
3. After thoroughly understanding the diverse identities and multi-stage practices of visitors in the museum, accurately position the profile of museum visitors. Enhance the museum’s ability to "create meaning and cultural output" by leveraging the cultural and symbolic aspects of the museum space and artifacts. Strengthen visitors’ cultural consumption satisfaction and enjoyment by emphasizing the practicality, innovation, quality, and diversity of cultural and creative designs. This strategy aims to increase visitors’ willingness to consume and expand cultural communication pathways and effects.
4. Prioritize the convenience and relevance of public visiting and consumption demands. In addition to utilizing various channels and media (social media, online marketing, cultural events) for museum promotion and marketing, establish museum introductions and location cues at major transportation hubs. Develop collaborative visiting experience projects to enhance the public’s willingness for museum visits, cultural learning, and broaden the scope of communication.

### Limitations and future research directions

The sampled institution in this study, the Nan Song Deshou Palace Relics Site Museum in Hangzhou, China, is a local museum established less than a year ago, designed to exhibit and convey the "specific historical stage and culture." Unlike comprehensive large-scale or national museums, such as the Palace Museum, its uniqueness lies in the concentrated exhibition of the Nan Song Dynasty site, artifacts, and culture. Consequently, this research aims to provide reference suggestions for similar museums in terms of functional construction, services, and cultural dissemination by conducting research and analysis on this museum and its current visitors. While this study reveals conclusions with certain representativeness and exploratory value, offering valuable insights for the improvement of functional settings, services, and communication strategies for the sampled museum and potentially for other museums of a similar nature, its applicability to other types of museums (e.g., art, science) and provincial or local comprehensive museums remains a topic for discussion and validation.

Furthermore, the sample size of this study consists of thirty-four interviewees. Despite efforts to ensure the representativeness and diversity of demographic characteristics in the sample, there is room for further expansion to examine the accuracy and effectiveness of the study’s conclusions, engaging in a dialogue with the results obtained in this research. Additionally, the cultural background, values, daily behavioral habits of museum visitors, and their experiences in visiting other cultural venues warrant further investigation regarding their impact on museum visits, cultural reception, revisit intentions, secondary communication intentions and behaviors [87].

Lastly, this study recognizes the implicit needs of museum visitors for "social presence, interaction, identification, and a sense of belonging." These needs manifest as a dual-sided feature involving both active establishment and passive acquisition. The study acknowledges the potential influence of the strength and weakness of social capital connections on individual well-being and behavior, questioning the pathways through which these effects occur [81]. Whether this influence has correlations with the social background and cultural education in the context of Chinese society is also an important topic for discussion.
